# Supplementary material for: Digital Transformation in Musculoskeletal Ultrasound: Acceptability of Blended Learning
Source: Diagnostics (Basel). 2023 Oct 20;13(20):3272. doi: 10.3390/diagnostics13203272 (PMC10606223; doi:10.3390/diagnostics13203272)
Supplement: Supplementary file 1 [file diagnostics-13-03272-s001.zip › diagnostics-2656803-supplementary.pdf]

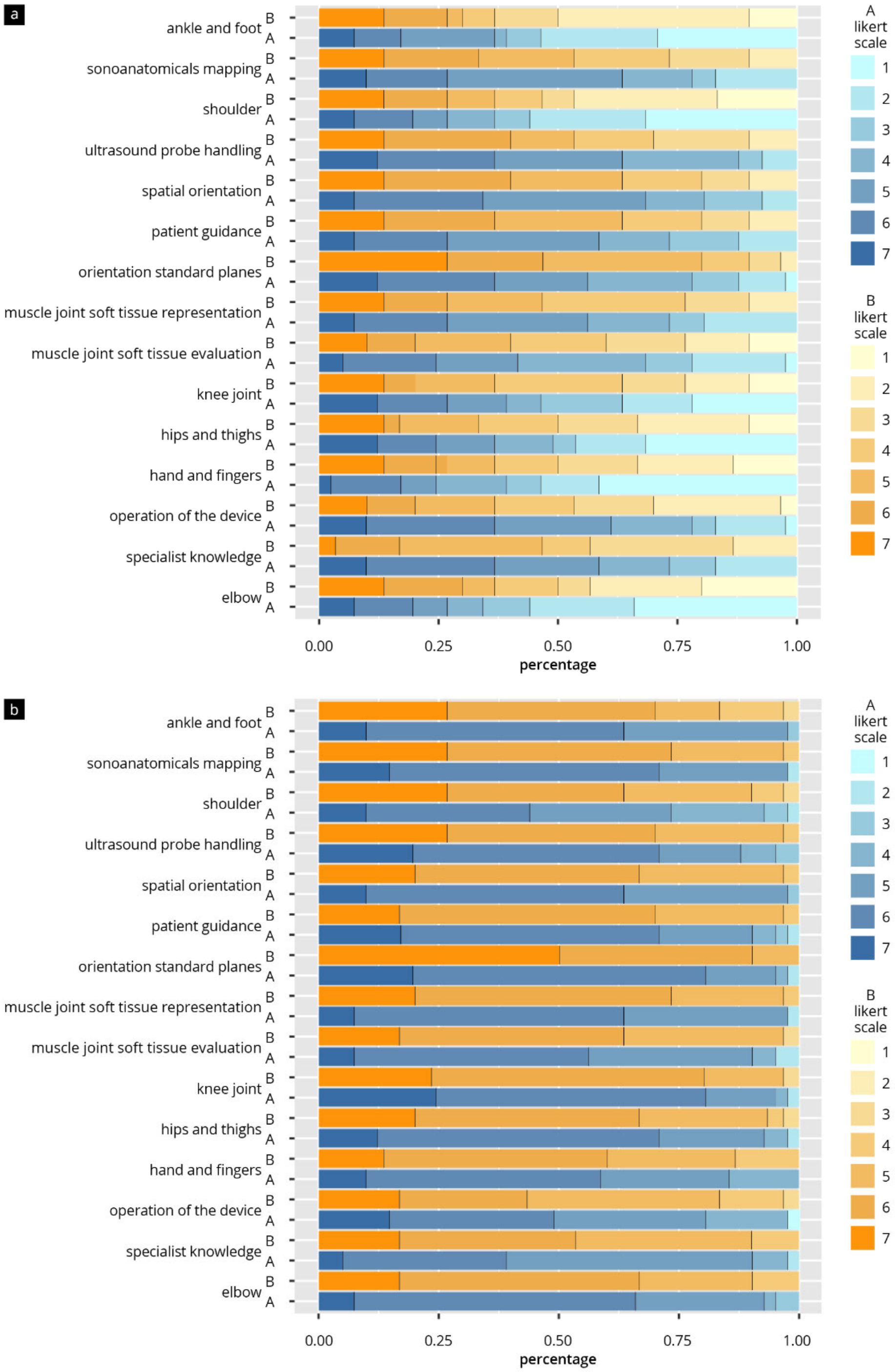

Figure S1. Evaluation Results of Course Model A (blue) and B (orange) in Regards to the Individual Items.

The bar plot shows the results of the "self-evaluation pre-course" (a) and "self-evaluation post-course" on a seven-point Likert scale.

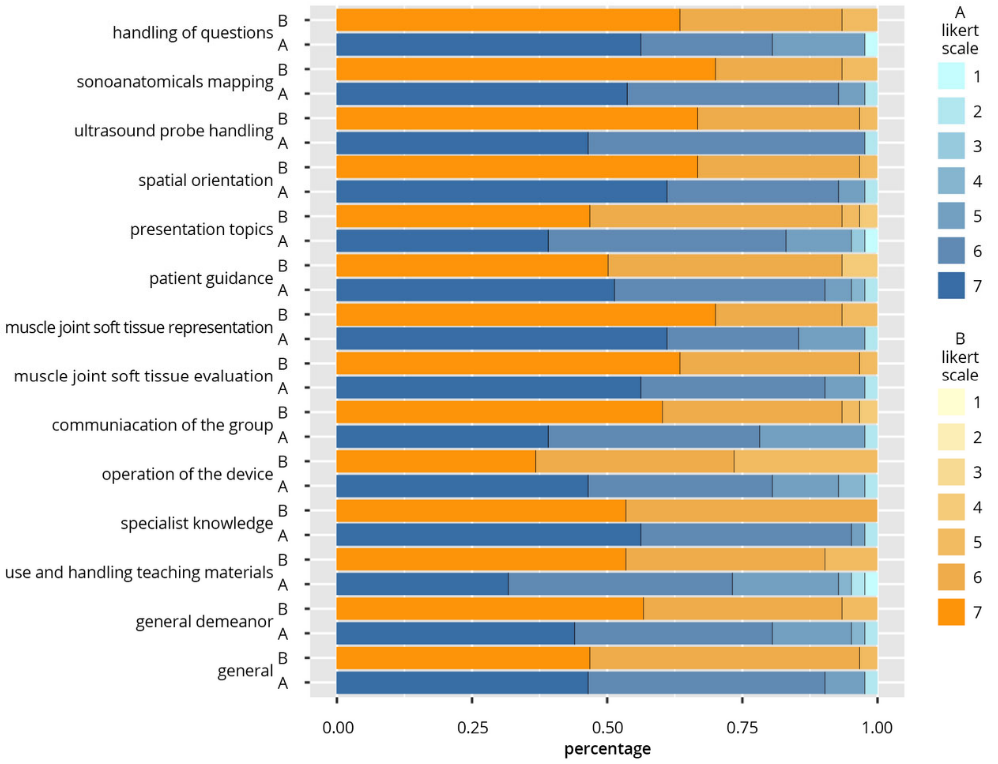

Figure S2. Evaluation Results of the Course Model A (blue) and B (orange) in regards to the individual items.

The bar plot shows the results of "Evaluation of the Tutor" on a seven-point Likert scale.

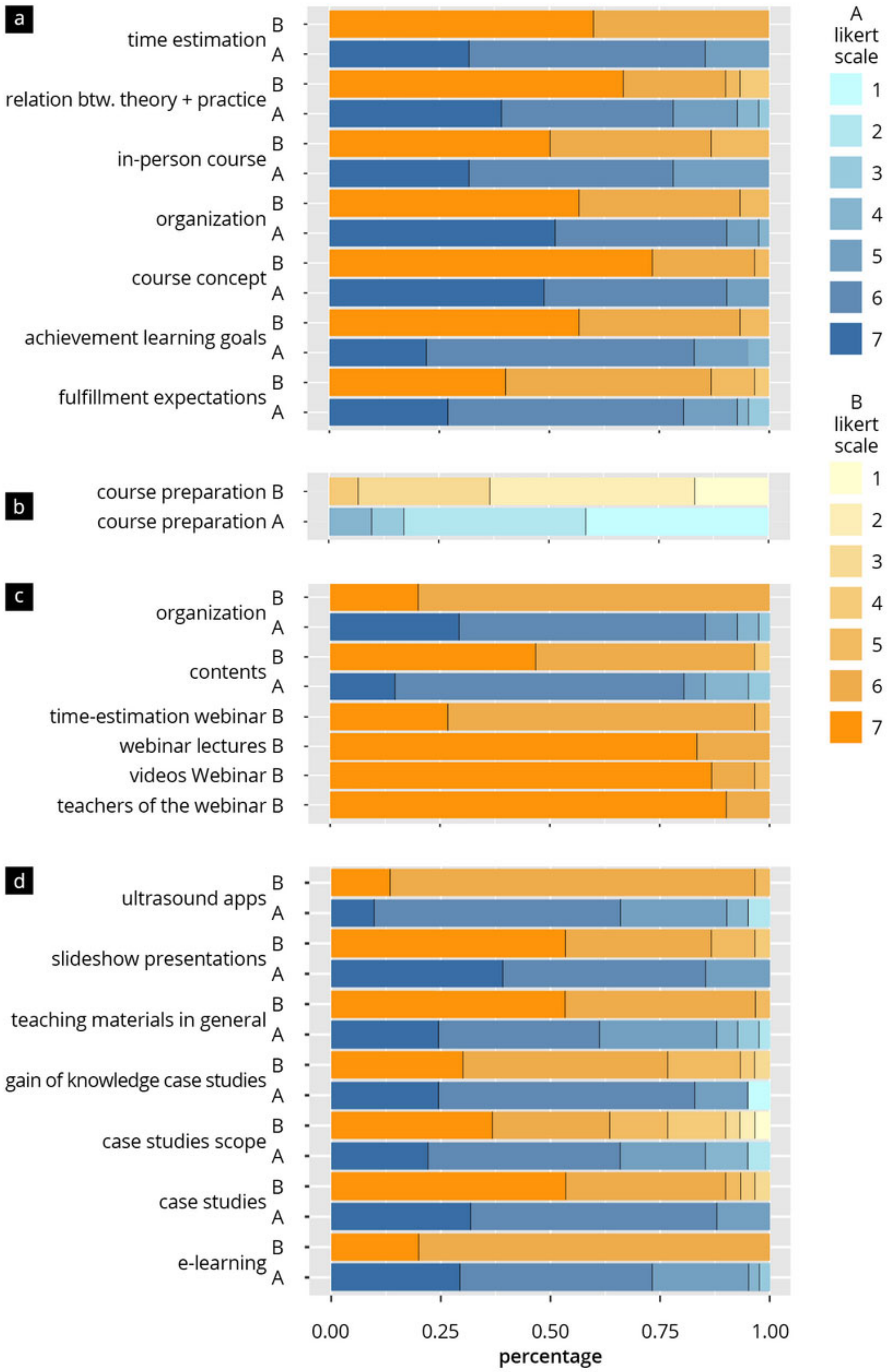

Figure S3. Evaluation results of the individual items of the questionnaire (A: blue, B: orange). The bar graph presents the percentage of responses given for the individual questionnaire items for the topics: (a) evaluation of the course, (b) course preparation, (c) evaluation of the webinar, (d) evaluation of the teaching material.

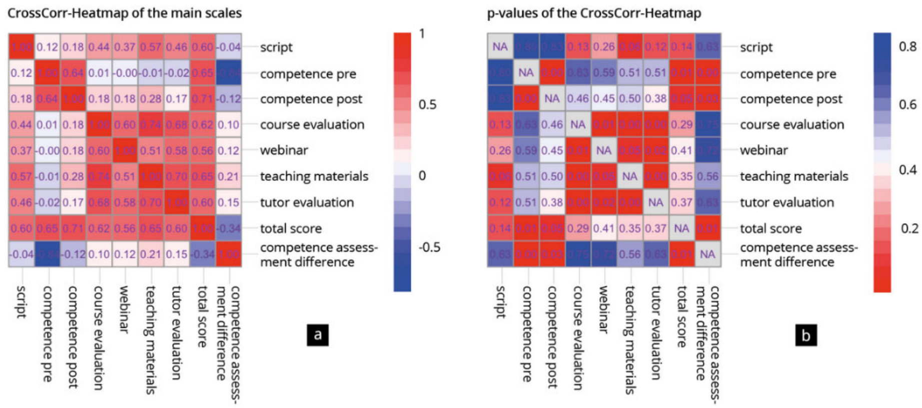

Figure S4. CrossCorr-Heatmap of the themes with gradients (a) and p-values (b).
